# Cost-effectiveness and economic returns of group-based parenting interventions to promote early childhood development: Results from a randomized controlled trial in rural Kenya

**DOI:** 10.1371/journal.pmed.1003746

**Published:** 2021-09-28

**Authors:** Italo Lopez Garcia, Uzaib Y. Saya, Jill E. Luoto

**Affiliations:** 1 RAND Corporation, Santa Monica, California, United States of America; 2 Pardee RAND Graduate School, Santa Monica, California, United States of America; Emory University, UNITED STATES

## Abstract

**Background:**

Early childhood development (ECD) programs can help address disadvantages for the 43% of children under 5 in low- and middle-income countries (LMICs) experiencing compromised development. However, very few studies from LMIC settings include information on their program’s cost-effectiveness or potential returns to investment. We estimated the cost-effectiveness, benefit–cost ratios (BCRs), and returns on investment (ROIs) for 2 effective group-based delivery models of an ECD parenting intervention that utilized Kenya’s network of local community health volunteers (CHVs).

**Methods and findings:**

Between October 1 and November 12, 2018, 1,152 mothers with children aged 6 to 24 months were surveyed from 60 villages in rural western Kenya. After baseline, villages were randomly assigned to one of 3 intervention arms: a group-only delivery model with 16 fortnightly sessions, a mixed-delivery model combining 12 group sessions with 4 home visits, and a control group. At endline (August 5 to October 31, 2019), 1,070 children were retained and assessed for primary outcomes including cognitive and receptive language development (with the Bayley Scales of Infant Development, Third Edition) and socioemotional development (with the Wolke scale). Children in the 2 intervention arms showed better developmental outcomes than children in the control arm, although the group-only delivery model generally had larger effects on children. Total program costs included provider’s implementation costs collected during the intervention period using financial reports from the local nongovernmental organization (NGO) implementer, as well as societal costs such as opportunity costs to mothers and delivery agents. We combined program impacts with these total costs to estimate incremental cost-effectiveness ratios (ICERs), as well as BCRs and the program’s ROI for the government based on predictions of future lifetime wages and societal costs. Total costs per child were US$140 in the group-only arm and US$145 in the mixed-delivery arm. Because of higher intention-to-treat (ITT) impacts at marginally lower costs, the group-only model was the most cost-effective across all child outcomes. Focusing on child cognition in this arm, we estimated an ICER of a 0.37 standard deviation (SD) improvement in cognition per US$100 invested, a BCR of 15.5, and an ROI of 127%. A limitation of our study is that our estimated BCR and ROI necessarily make assumptions about the discount rate, income tax rates, and predictions of intervention impacts on future wages and schooling. We examine the sensitivity of our results to these assumptions.

**Conclusions:**

To the best of our knowledge, this study is the first economic evaluation of an effective ECD parenting intervention targeted to young children in sub-Saharan Africa (SSA) and the first to adopt a societal perspective in calculating cost-effectiveness that accounts for opportunity costs to delivery agents and program participants. Our cost-effectiveness and benefit–cost estimates are higher than most of the limited number of prior studies from LMIC settings providing information about costs. Our results represent a strong case for scaling similar interventions in impoverished rural settings, and, under reasonable assumptions about the future, demonstrate that the private and social returns of such investments are likely to largely outweigh their costs.

**Trial registration:**

This trial is registered at ClinicalTrials.gov, NCT03548558, June 7, 2018. American Economic Association RCT Registry trial AEARCTR-0002913.

## Abstract 2

Italo Lopez Garcia and colleagues conduct an economic evaluation of an effective parenting intervention to promote cognitive, receptive language, and socioemotional development among children under 3 years in Kenya.

## Author summary

**Why was this study done?:**

- It is estimated that 43% of children under 5 years of age from low- and middle-income countries (LMICs) are at risk of not reaching their full developmental potential as adults due to poverty, lack of stimulation, and nutritional deficiencies.
- Numerous early childhood development (ECD) parenting interventions that teach parents strategies to improve responsive stimulation and nutritional practices with children have demonstrated that they can help improve children’s outcomes.
- Yet very few ECD parenting studies have shared information about their program’s costs to enable an analysis of its cost-effectiveness or potential economic returns, which is crucial to inform policy decisions on whether to invest in ECD parenting programs.
- Among the very few studies that report a program’s cost-effectiveness, none has accounted for the opportunity costs that program participants must incur to attend a program and enact any recommended behavioral changes.

**What did the researchers do and find?:**

- We calculate the total costs, cost-effectiveness, and compare the benefits versus costs for an ECD parenting intervention recently tested across 60 villages in rural western Kenya involving nearly 1,200 mothers and their young children.
- The program compared 2 different delivery models, one featuring only group visits within villages and a second featuring a combination of group and home visits. Both were delivered by local community health volunteers (CHVs) over 8 months.
- We estimate the opportunity costs for the CHVs and participants in our program under both delivery models and predict the future benefits and costs stemming from participation, such as increases in lifetime wages and schooling costs.
- We find that children experienced large improvements in their cognitive development at very reasonable costs under both delivery models, but the group-only model was the most cost-effective.
- Under reasonable assumptions about the future, we find that long-term program benefits could outweigh its associated costs by a factor of 15, implying a large potential return on investment (ROI) for a government willing to scale this program.

**What do these findings mean?:**

- Our findings are very encouraging for the investment into ECD parenting programs for resource-poor and rural settings.
- As national and international policymakers increasingly encourage investment into ECD parenting programs, our results represent a strong case study for a promising means to do so.

## Introduction

Scientific evidence confirms that the first 3 years of life are when risk factors such as inadequate parental stimulation exert the greatest harm on child development and when effective interventions can have the greatest benefit [1,2]. Numerous studies have demonstrated the effectiveness of parenting interventions that promote responsive stimulation to improve early childhood development (ECD) in low- and middle-income country (LMIC) settings among children under 3 years of age [3,4]. However, very few studies include accompanying economic evaluations to provide information about a program’s cost-effectiveness or the potential economic returns to scaling these interventions in resource-limited settings [5,6]. For example, while recent meta-analyses have identified more than 40 studies evaluating early childhood stimulation interventions targeted to young children in LMICs [4,7], another recent meta-analysis of economic evaluations of ECD parenting interventions and programs identified just 5 studies that provided information about program costs [8]. Institutions such as the World Health Organization (WHO), United Nations International Children’s Emergency Fund (UNICEF), and the World Bank now advocate filling this gap via collection and sharing of program cost data from evaluations of ECD interventions in LMIC settings [9].

Data on program costs can enable different types of economic evaluations of ECD interventions. Cost-effectiveness analysis (CEA) compares the relative costs of a program to its generated impacts on outcomes and is one of the most important indicators used to inform scalability [10]. However, existing CEAs of ECD interventions from LMICs are more readily available for nutrition or health interventions or for preschool programs focused on older ages, with less attention paid to responsive parenting interventions for young children [4,8,11]. Moreover, to date, the very few parenting interventions including a CEA adopt a provider’s costing perspective, thereby excluding any societal costs such as opportunity costs to participants and delivery agents. This risks creating perverse incentives for governments to offload such costs onto users and goes against recommended best practice [12]. Notably, no CEA studies are available from sub-Saharan Africa (SSA), where the coverage of ECD programs for young children is lowest, and supporting evidence to expand coverage of responsive parenting interventions is arguably most needed [10].

A more comprehensive economic evaluation may also include a benefit–cost analysis (BCA), which compares the monetized expected program benefits relative to its costs, and can better inform policy on the potential benefits to society of scaling a program. Yet BCAs are even more rare than CEAs in the evaluation of ECD parenting programs in LMICs [13], likely because it can be difficult to estimate long-term benefits without making a number of assumptions (or waiting many years to measure those benefits). A recent systematic review identified only 3 evaluations of ECD parenting programs focused on young children from LMICs that provided benefit–cost ratios (BCRs) [8]. Again, none are from SSA.

In addition to a lack of economic evaluations of ECD parenting programs, there has been growing interest in understanding the delivery format that is most scalable to reach young children before age 3 in LMICs [14,15]. ECD parenting programs that have been shown to be effective are typically delivered in one of 2 formats: individual home visits or group sessions held in the village or at local health clinics. Home visits have the advantage of offering personalized coaching, demonstration, and feedback, but are time and labor intensive and hence expensive to deliver at scale. Group sessions are less labor intensive and may be relatively less expensive to deliver, can encourage peer support, and can help modify group norms for child rearing, but may be weak to overcome personal barriers to behavioral change [3]. Knowing the cost-effectiveness of competing delivery models can help policymakers select the model that is most scalable for the local context.

The aims of this study are to perform an economic evaluation of an effective ECD parenting intervention targeted to families with children under age 3 in SSA that can contribute new insights about the relative cost-effectiveness of 2 different delivery models and to estimate their potential BCRs and returns to investment.

## Materials and methods

### Background of research trial

The intervention, named Msingi Bora (“Good Foundation” in Swahili), was a group-based parenting intervention implemented among rural Kenyan households with children aged 6 to 24 months at baseline that significantly improved child cognitive, receptive language, and socioemotional development among treated families [15]. The cluster randomized controlled effectiveness trial of Msingi Bora was conducted across 60 rural villages in western Kenya in the subcounties of East and South Rachuonyo within Homa Bay county and Sabatia subcounty within Vihiga county. The trial featured 3 study arms, each 20 villages in size: (1) a group-only delivery model with 16 fortnightly group sessions; (2) a mixed-delivery model combining 12 group sessions with 4 home visits; and (3) a control group that received no program under the status quo. Eligible participants within villages were mothers or other female primary caregivers aged 15 and over with a child between 6 and 24 months at recruitment without signs of severe mental or physical impairment. All participants provided written informed consent at the time of data collection. At baseline (October 1 to November 12, 2018), we collected household sociodemographic information and measures of child developmental outcomes from 1,152 eligible households. The 60 villages were then randomly assigned to one of the 3 study arms. The intervention lasted from mid-November 2018 to mid-July 2019 and was immediately followed by an endline survey (August 5 to October 31, 2019) among 1,070 retained households. The primary endpoints were child cognitive and language development as measured using the Bayley Scales of Infant Development, Third Edition [16] and child socioemotional development measured with the Wolke Scale [17]. Ethics approval was obtained from the ethics committee at Maseno University in Kisumu, Kenya and RAND; the trial is registered with ClinicalTrials.gov NCT03548558, and the study protocol has been published [18].

Delivery agents were drawn from the existing cadre of local community health volunteers (CHVs), which is a part-time and voluntary position under Kenya’s Ministry of Health, tasked with improving community health through home visits. For our study, CHVs received a total of 16 days of training, with 8 days before the program started to cover the first half of sessions (sessions 1 to 8) and another 8 days at midline to cover the second half of sessions (sessions 9 to 16). Each training included 5 days in the city of Kisumu to introduce the material and curriculum, followed by 3 days of supervised practice in the rural subcounties. From session 4 onwards, CHVs received monthly 1-day refresher trainings in their subcounties to prepare them for that month’s upcoming sessions.

Mothers and children were invited to attend every session and received a small gift for attendance (e.g., small bar of soap or bag of milk). Every fourth session served as a review session, for which households in the group-only arm continued with group meetings, while households in the mixed arm received individual home visits. During these home visits, participants received identical messages to those in the group reviews, but the focus was personalized on that family. The remaining 12 non-review sessions were delivered in group meetings in both intervention arms. Group sessions took place in local community centers or churches and lasted a median of 90 minutes, while the median home visit lasted an hour. The median travel time for CHVs to group meetings was 20 minutes each way, while the median travel time to each home visit was also 20 minutes. Over the 8-month program, mothers in the group-only arm averaged 64% attendance versus 74% in the mixed-delivery arm (Table 1), where this difference was driven only by higher attendance (88%) to the 4 review sessions that were delivered as home visits in the mixed-delivery arm (and thus, mothers did not have to travel to these sessions) [19].

**Table 1:** Background information on the intervention arms and control group.

Intervention arm	Group-only delivery model	Mixed-delivery model	Control
**Number of CHVs and villages**	20	20	20
**Number of children planned at enrollment**	400	400	400
**Program delivery strategy**	12 non-review group sessions held fortnightly and 4 group review sessions; monthly 1-day refresher trainings for CHVs were performed in each subcounty for that month’s sessions	12 non-review group sessions held fortnightly and 4 review sessions delivered through home visits. In these home visits, CHVs delivered identical content to the group review sessions, but the focus was personalized on that family. CHVs visited each participant household during the same week that a group review session was held in group-only villages	No sessions (only received a flyer on child feeding during the baseline survey that was made available to all respondents in all study arms)
Intervention impacts^*^ Cognition Receptive language Socioemotional	Effect size/95% CIs: 0.52 (0.21–0.83) 0.42 (0.08–0.77) 0.23 (0.03–0.44)	Effect size/95% CIs: 0.34 (0.05–0.62) 0.20 (−0.11–0.52) 0.22 (0.05–0.38)	
**Average attendance**	64%	74%	
**Median session duration**	90 minutes (1.5 hours) for group sessions	90 minutes (1.5 hours) for group sessions; 60 minutes (1 hour) for home visit review sessions	
**Median travel time to group meetings**	20 minutes each way (0.66 hours roundtrip)	20 minutes each way (0.66 hours roundtrip)	
**Median travel time to home visits**	NA	20 minutes to each home visit and extra 20 minutes each day to return home (with each CHV doing 4 home visits per day)	
**% group non-review sessions supervised**	214/240 (89%)	209/240 (87%)	
**% review visits supervised**	73/80 group review sessions supervised (91%)	122/1,531 home visits supervised (8%)	

*Results based on a final sample of *N* = 1,070 at endline (346 in group, 373 in mixed, and 351 in control arms) based on ITT analyses of each intervention arm versus comparison arm as presented in Luoto and colleagues [15]. Median time estimates are based on monitoring data and CHV self-assessment forms completed after each session.

CHV, community health volunteer; ITT, intention to treat.

The local nongovernmental organization (NGO) Safe Water and AIDS Project (SWAP) oversaw the study’s implementation. Three of SWAP’s staff from the central office in Kisumu provided oversight of the project and became lead trainers of CHVs under a Training of Trainers (ToT) model. They also coordinated the work of 3 subcounty supervisors who were hired by the research project to observe and monitor each group session and at least 1 home visit from each review session in the assigned villages. Subcounty supervisors were assisted by 1 or 2 “mentor” CHVs who had been previously trained in a piloting phase to deliver the program across 6 villages not included in the main trial. In the group-only arm, 89% of the non-review and 91% of the review sessions were supervised. In the mixed arm, 87% of the group non-review sessions and 8% of the 1,531 attempted home visits were supervised (Table 1). Supervisors rated CHVs using monitoring forms with a checklist of items related to fidelity and quality of delivery. CHVs were provided supervisor feedback immediately after each session and filled out self-evaluation forms after each session.

### Outcomes

The impact analysis of Msingi Bora was by intention to treat (ITT) among the 1,070 children assessed at endline [15]. We estimated adjusted multivariate linear regressions of each age-standardized outcome on treatment assignment relative to the control group. Preplanned adjustments included child age, sex and birth order, maternal education, household wealth, baseline outcomes (if available,) and subcounty strata fixed effects. We found that both the group-only and the mixed-delivery models significantly improved child cognitive and socioemotional development relative to the control group, and the group-only model also showed statistically significant improvements in child receptive language. The ITT effect sizes of improvements in the group-only arm were 0.52 standard deviation (SD; *p* = 0.001) in cognition, 0.42 SD (*p* = 0.017) in receptive language, and 0.23 SD (*p* = 0.024) in socioemotional development. In the mixed-delivery arm, the ITT effect sizes were 0.34 SD (*p* = 0.021) in cognition, 0.20 SD (*p* = 0.17) in receptive language, and 0.22 SD (*p* = 0.011) in socioemotional development (Table 1) [15]. Although the mixed-delivery arm had smaller effect sizes on child cognition and receptive language, differences across intervention arms were only statistically significant based on 1-sided tests at the 10% level.

### Economic evaluation

This study is reported as per the Consolidated Health Economic Evaluation Reporting Standards (CHEERS) guideline (S1 Checklist) [20]. We estimate incremental cost-effectiveness ratios (ICERs), BCRs, and return on investment (ROI) for the 2 delivery models tested in the Msingi Bora trial. The CEA follows a preanalysis plan laid out in our study protocol [18] to calculate the incremental ITT impacts on child developmental outcomes divided by the total present period costs of an intervention (either group only or mixed delivery), where both are expressed relative to the control group. Following recent guidelines for cost-effectiveness analyses [21,22], we present ICERs in terms of SD improvements in child developmental outcomes per US$100 investment. In addition to the preplanned CEA, we also estimate the expected BCRs and ROIs of our program to help fill this gap in the literature evaluating ECD interventions and programs in LMICs.

All costs and benefits in local currency included in our analyses were converted at 101.8 Kenyan Shillings = US$1 and reported in 2020 prices, adjusted for inflation.

### Costing perspective

For the calculation of cost-effectiveness, we adopt a societal perspective that combines the implementation costs a health service provider would incur if the program were to be scaled using local CHVs with present period societal costs to the household and the community. These societal costs include the opportunity costs of mothers and CHVs to travel to and attend sessions, as well as rental costs for venues to host the sessions if operating at scale (although we did not pay for venues in our trial). We use accounting cost methods for the provider costs and economic costing methods to incorporate the opportunity costs of participation for mothers and CHVs. For the calculation of BCRs and ROIs, we additionally incorporate the predicted future costs and benefits stemming from gains in cognitive skills following participation in the program. Future costs include the costs of increased schooling attendance among participants in a scaled version of the program [23], and future benefits include the expected gains in lifetime wages. Research costs (e.g., piloting, qualitative and quantitative data collection, and cost of researchers conducting the trainings) were excluded.

### Costs

Provider costs relevant to implementing the Msingi Bora program were collected during the 8-month intervention period using quantitative financial data reported by SWAP. For these costs, we used a step-down accounting costing methodology using actual program costs rather than budgeted costs [24,25] and where costs were divided into direct and indirect implementation costs depending on whether they were related to specific implementation activities or not. All program-related cost data were monitored on a regular basis during the start-up and intervention period by SWAP’s financial manager. We used economic costing methods to estimate opportunity costs for CHV and mother participants. Travel times to conduct sessions and session duration both for home visits and group meetings were based on CHV self-assessment forms collected after every session. We used supervisor monitoring forms to impute supervision time-related costs.

Unit costs by intervention arm are presented in Table 2.

**Table 2:** Unit costs of Msingi Bora.

	Unit	US$
**Direct costs**		
*Personnel*		
CHV stipend	$/month-person	20
Mentor CHV stipend	$/month-person	20
Subcounty supervisor wage	$/month-person	550
SWAP staff average wage	$/month/person	780
*Time use costs*		
CHV opportunity cost (assumed wage rate)	$/hour	US$0.58
*Travel and accommodation*		
Centralized training: full board lodging	$/person-night	46
Centralized training: transportation allowance	$/person	10
Monthly subcounty training: transportation allowance	$/person-day	5
Monthly subcounty training: meals	$/person-day	13
Supervision: supervisors’ transport allowance	$/person-week	[25–50]
*Food and supplements*		
Incentives for participants (soap, milk, or eggs)	$/unit	0.2
Picture book given to participants	$/unit	3.0
**Indirect costs**		
Start-up costs (online data transfer and storage)	$ (one time)	1,360
**Societal costs**		
Venue hire for group sessions	$/session	5
Mother’s opportunity cost of time (assumed wage rate)	$/hour	0.19

The centralized trainings took place in Kisumu for which all staff were provided transportation allowance in addition to full board lodging. Supervisors’ transport allowance varied based on subcounty location. SWAP staff average wage is a weighted average based on time commitments to the project across the supervisory or training staff. CHV opportunity cost is based on KIHBS data on mean wages for workers who work outside the home at least 20 hours per week from Vihiga and Homa Bay counties, updated to 2020 prices. Mother’s opportunity cost is based on the mean female wage from KIHBS data from Vihiga and Homa Bay counties updated to 2020 prices. All costs used for calculations were originally in Kenyan Shillings converted to USD using exchange rate of 1 USD = KSh 101.8 as of January 2020.

CHV, community health volunteer; KIHBS, Kenyan Integrated Household Budget Survey; SWAP, Safe Water and AIDS Project.

#### Provider costs

We present the costs that were required to implement the intervention “as it happened,” which include additional costs incurred due to unexpected delays in fieldwork, as well as cost inefficiencies in practice (e.g., having more than 1 supervisor at a given session). The only exception is that we account for the higher time use costs for CHVs delivering home visits for the 4 review sessions in the mixed-delivery arm using estimates of the opportunity cost of their time. For the sake of comparison, in S2 Text, we present an alternative “best case” costing scenario based on our calculations of program costs without those delays and inefficiencies that better reflect how the program was intended to be implemented and thus how much it would arguably cost to replicate it in similar settings taking advantage of potential economies of scale. Results from this comparison are included in a sensitivity analysis.

#### Direct program costs

Direct costs included salaries and stipends for personnel (CHVs, mentor CHVs, subcounty supervisors, and SWAP staff), training, travel and accommodation for training, and any food and supplements necessary for the implementation of the program.

**Personnel**

We include the flat monthly stipends paid to CHVs and mentor CHVs, as well as salary costs for 3 subcounty supervisors and 4 SWAP staff members involved in day-to-day activities of implementation, supervision, and training, prorated to their percentage time commitment to the project. These base salary costs were equally shared among the 2 intervention arms. For the 4 review sessions that were delivered through home visits in the mixed-delivery arm, we add to these salary costs the time use costs that CHVs, mentor CHVs, and subcounty supervisors spent delivering or supervising these sessions using an estimated hourly wage rate and the time taken to conduct these activities (Table 1). These time use costs were larger in the mixed-delivery arm because home visits are more time intensive than group meetings. To estimate the opportunity costs of CHV’s time, we use the 2015 to 2016 Kenyan Integrated Household Budget Survey (KIHBS), which provides individual data on wages at the county level. We calculate the average implied hourly wage for all workers who work at least 20 hours per week in some outside paid activity in the study counties of Homa Bay and Vihiga, updated to 2020 prices. We used session monitoring data to impute supervision costs for mentors and supervisors as appropriate.

**Travel and accommodation**

These costs include transportation, meals, and lodging for CHVs, mentor CHVs, and subcounty supervisors to attend the 2 centralized 5-day training sessions in Kisumu, transportation costs for supervisors to supervise sessions, and transportation and meal costs for mentor CHVs and CHVs to attend the monthly refresher trainings hosted in the respective subcounties. These costs again differ by delivery arm due to the travel time necessary to conduct home visit review sessions in the mixed-delivery arm, again priced at local wage rates.

**Supplements and incentives**

We include milk, soap, or eggs given to participants during sessions, as well as a locally published picture book given to all children in session 14.

#### Indirect program costs

These costs included start-up costs such as the cost of tablets used for session monitoring and an online data transfer platform, administrative costs such as the design and printing of manuals for CHVs used during training, mobile phone credit for CHVs, mentor CHVs, and supervisors, and the printing of T-shirts for CHVs and certificates for participants who completed the program.

#### Societal costs

In addition to the provider costs above, we include the following societal costs.

**Mothers’ opportunity cost of time**

Mothers who participate in Msingi Bora pay opportunity costs of their time stemming from attending sessions, as well as enacting the new recommended behaviors with their children at home. To price this time, we again use the 2015 to 2016 KIHBS to estimate an implied hourly wage for all mothers based on the average wage rate for females in the 2 study counties, regardless of their employment status, and updated to 2020 prices (Table 2). We combine average attendance, session duration, and travel times from Table 1 to estimate the time mothers spent attending sessions, and then in response to a reviewer recommendation, we add to this an estimated 1 additional hour per week in stimulation practices over 3 years of the child’s life to reflect time opportunity costs associated with maternal behavioral changes. This implicitly assumes no opportunity costs for behavioral changes after 3 years, but aligns with Kenya’s 2018 policy of universal free preschool beginning at age 3 [26]. Moreover, our curriculum emphasized that mothers can stimulate their children while doing their daily chores such as speaking to the child and providing the child with a playing object and did not ask for more than 10 minutes per day of direct stimulation activities.

**Venues**

While our program enjoyed the free use of community centers or churches for the group-based sessions, under a scaled version of Msingi Bora, we estimate that venues would no longer be free and thus include a daily rental fee per venue per group session, for which there are 12 in the mixed-delivery arm and 16 in the group-only arm (Table 2).

### CEA

We calculate incremental cost-effectiveness separately for each delivery model as the ratio of that intervention model’s ITT impact on each child outcome divided by that model’s total present period costs, where we use a societal perspective and present results in 2020 US$. This allows us to interpret our results in terms of SD improvement in a given outcome per US$100 investment by intervention arm. Following standard recommendations for reporting of cost-effectiveness [12], we also present ICERs based on provider costs, although we only use these to facilitate comparisons with existing estimates. Standard errors (SEs) for our estimates are recovered from simple linear transformations of SEs of the intervention impacts, which we assume to be normally distributed random variables.

### BCA

To estimate BCRs and ROIs of our program in the long term, we additionally consider future costs and benefits that would result from increased cognitive abilities among participants in a scaled version of our program. Future costs include additional schooling costs due to increased attendance, and future benefits include net gains in lifetime wages.

#### Schooling costs

Improvements in child cognition resulting from the intervention may increase schooling attendance and associated costs such as additional infrastructure, tuition payments, and teacher salaries. To predict these future schooling costs, we first predict the intervention impacts on schooling attendance, which we obtain as the product of our intervention impacts on cognition with an estimate for the additional years of schooling associated with an increase in cognitive abilities. We estimate this schooling return to cognition using data from the Kenya Life Panel Survey (KLPS) rounds 1 to 4 (2003 to 2005, 2007 to 2009, 2011 to 2014, and 2018 to 2019) [27,28], which includes longitudinal data on cognitive tests, schooling, earnings, and household sociodemographics for a representative sample of more than 5,000 individuals participating in the Primary School Deworming Project (PSDP) between 1997 and 2001 in nearby Busia District. Using multivariate linear regressions of years of schooling on an age-standardized measure of cognition, we estimate that a one SD increase in cognition is associated with an increase of 1.79 (SE = 0.12, *p* < 0.0001) years of schooling after adjusting for covariates including parental education, survey wave and month of interview, a female indicator variable, and baseline 1998 school grade fixed effects (more details in Table A in S1 Text). Combined with our ITT impacts on cognition (Table 1), we predict that participation in our program would increase schooling by 0.93 years in the group-only arm and by 0.61 years in the mixed-delivery arm. We estimate final schooling costs for each delivery model by multiplying these predicted intervention impacts on schooling with the public cost of an additional grade per child per year, which we assume to be 15% of the basic wage for low-skilled workers from KIHBS wage data, following Nandi and colleagues [29].

#### Gains in lifetime wages

To predict the expected gains in lifetime wages from our intervention, we need 2 ingredients: (a) data on wage profiles by age of children as adults for our setting; and (b) the predicted intervention impacts on wages. We obtain potential wage profiles by age of children as adults from a representative sample of individuals aged 16 to 64 again using the 2015 to 2016 KIHBS. We restrict the sample to at least halftime workers (working at least 20 hours per week in any paid occupation) and calculate the life stream of average earnings by age expressed in 2020 prices, as well as the sum of discounted earnings to present values adjusting for expected survival probabilities using age life tables from Kenya [30]. To predict the intervention impacts on wages, we combine our ITT impacts on cognition with an estimate for the wage return to increased cognitive abilities, which we estimate with KLPS data. Using multivariate linear regressions of log wages on an age-standardized measure of cognition, we predict that a one SD increase in cognition is associated with a 39.7% (SE 0.058, *p* < 0.0001) increase in annual wages after controlling for the same covariates as the schooling regressions (more details in S1 Text). Combined with the intervention effects on cognition reported in Table 1, we estimate that the intervention would increase annual long-term wages by 20.6% in the group-only arm and by 13.5% in the mixed-delivery arm. Net gains in lifetime earnings by arm are the product of the sum of discounted earnings, and these predicted intervention effects on adult wages.

### Discounting

For our BCA, we follow standard practice to translate the long-term costs and benefits of our program into present discounted value (PDV) terms using an annual discount rate to reflect their delayed nature. We assume an annual discount rate of 5%, which is in line with previous benefit–cost analyses of ECD programs from LMIC settings [31–33]. However, in a sensitivity analysis, we examine how our BCR and ROI estimates would change under different assumptions about the discount rate.

#### Benchmark BCR and ROI calculations

We use our estimates of the full societal benefits and costs that includes these future schooling costs and wage increases to estimate both a BCR and the expected ROI. The BCR compares the child’s expected increase in discounted lifetime earnings resulting from early cognitive gains following participation in Msingi Bora versus its average costs per child, also expressed in PDV terms. The ROI is the difference in additional expected tax revenues due to the increased PDV of lifetime earnings from the intervention relative to the average costs per child, expressed in terms of a percentage change. For calculating ROI, we assume a 14.6% average tax rate, again based on KIHBS earnings data updated to 2020 prices and matched to Kenya’s marginal tax rate schedule from 2020 [34]. In a sensitivity analysis, we explore alternative average tax rates. Positive ROIs indicate that public revenues are greater than the costs of scaling the program.

In order to derive SEs for our estimated BCRs and ROIs, we perform 1,000 Monte Carlo simulations that allow for uncertainty in our estimates of wage and schooling returns to cognitive abilities, assuming that all these parameters are normally distributed random variables.

#### Sensitivity analyses

We conduct 2 types of sensitivity analyses. We first test for the robustness of our BCRs and ROIs by systematically varying one parameter at a time while holding all others constant. This allows us to identify what “extreme values” are needed for our BCR to be less than 1 and our ROI less than 0, which, in turn, would make scaling Msingi Bora a poor societal investment. We vary the discount rates, total costs per child, average tax rate, cognitive wage returns, and the public cost of an additional grade per child per year to find these extreme values. In addition to these parameters, we test for the degree of fade-out in impacts in the longer-term that our program could sustain while still maintaining positive net benefits and ROIs, which is motivated by a recent systematic review of ECD parenting trials from LMICs that finds that early intervention impacts on children’s developmental outcomes tend to fade over time [35].

In addition, in Table B in S2 Text, we estimate by how much the resulting ICERs, BCRs, and ROIs would change if we allow for both socioemotional and cognitive abilities to influence lifetime wages and if we use costs based on our “best case” scenario for provider costs that allows for some implementation efficiencies and economies of scale. For this analysis, we assume that a one SD increase in socioemotional skills leads to a 6.4% increase in wages as based on a recent study on the returns to socioemotional skills for Kenya [36].

## Results

### Program costs

The total costs for the program were US$56,171 for the group-only arm and US$58,103 for the mixed-delivery arm (Table 3), which translate into a total cost per child of US$140 in the group-only arm and US$145 in the mixed-delivery arm. Provider costs comprised the bulk of these total costs, amounting to US$47,423 in the group-only arm (US$119 per child) and US$48,670 in the mixed-delivery arm (US$122 per child). The only difference in these costs across the 2 models of delivery comes from the time use costs associated with the review sessions that account for CHVs spending an average of 18.5 additional hours delivering home visit review sessions relative to group review sessions. Travel and accommodation were by far the highest direct provider costs, largely as a result of the 2 centralized full board trainings. The small difference in these costs across the 2 delivery models stem from the extra travel time required for home visits, which we estimate at an additional 8.5 hours per CHV per review session in the mixed-delivery arm.

**Table 3:** Total costs of Msingi Bora.

Delivery model	Group only	Mixed delivery
***Direct provider costs***	**US$42,693**	**US$43,940**
Personnel: CHVs	US$3,570	US$4,230
Personnel: mentor CHVs	US$310	US$314
Personnel: subcounty supervisors	US$9,094	US$9,153
Personnel: implementing partner staff	US$7,271	US$7,271
Travel and accommodation	US$19,720	US$20,243
Supplements and incentives	US$2,729	US$2,729
***Indirect provider costs***	**US$4,730**	**US$4,730**
Administrative costs	US$3,301	US$3,301
Start-up costs	US$1,429	US$1,429
**Total provider costs**	**US$47,423**	**US$48,670**
**Provider cost per child**	**US$119**	**US$122**
**Present period societal costs**		
Mother’s opportunity costs	US$7,096	US$8,194
Venue costs	US$1,652	US$1,239
**Total present period societal costs**	**US$8,748**	**US$9,433**
**Present period societal cost per child**	**US$22**	**US$24**
**Total costs**	**US$56,171**	**US$58,103**
**Total cost per child**	**US$140**	**US$145**

Present period societal costs exclude long-term costs such as additional costs of schooling from cognitive gains. All costs originally in Kenyan Shillings converted to USD using exchange rate of 1 USD = KSh 101.8 as of January 2020. Costs include items as described in the Materials and methods section.

CHV, community health volunteer.

The present period societal costs were US$8,748 in the group-only arm (US$22 per child) and US$9,433 in the mixed-delivery arm (US$24 per child) (Table 3). This small difference stems from a few countervailing factors. On the one hand, the group-only arm entails 4 extra group review sessions that require greater venue costs, as well as greater time for mothers to travel and attend group sessions, which lasted 30 minutes longer than home visits on average. On the other hand, only mothers who attend sessions can enact the recommended behavioral changes, which implies greater mother opportunity costs on average due to the higher attendance at the 4 home visit review sessions in this arm.

### Cost-effectiveness

Table 4 reports the ICERs for each delivery model and considers costs under both provider and societal perspectives as reported in Table 3. Higher ratios imply a higher impact on child outcomes per US$100 invested. ICER estimates for the group-only arm are generally greater than those for the mixed-delivery arm due to the higher estimated impacts on child outcomes at slightly lower costs. For the group-only delivery model under a societal perspective, an additional US$100 investment is estimated to lead to a 0.37 SD improvement in child cognition, 0.30 SD improvement in receptive language, and 0.16 SD improvement in socioemotional development. Under the mixed-delivery model, the corresponding estimates are improvements of 0.23 SD, 0.14 SD, and 0.15 SD, respectively. ICERs under a provider perspective are slightly larger as societal costs are excluded from the denominator.

**Table 4:** Cost-effectiveness of Msingi Bora.

Cost-effectiveness ratios (additional SD per US$100)	Group only delivery	Mixed delivery
**Provider costs only**		
Cognition	0.44 (0.133)	0.28 (0.120)
Receptive language	0.35 (0.148)	0.16 (0.086)
Socioemotional	0.19 (0.088)	0.18 (0.069)
**Total societal costs**		
Cognition	0.37 (0.113)	0.23 (0.100)
Receptive language	0.30 (0.125)	0.14 (0.072)
Socioemotional	0.16 (0.074)	0.15 (0.058)

ICERs expressed as SD improvements per US$100 investment. SEs in parentheses are obtained from SEs of intervention impacts using a linear transformation of normally distributed random variables. All costs used for calculations were originally in Kenyan Shillings converted to USD using exchange rate of 1 USD = KSh 101.8 as of January 2020.

ICER, incremental cost-effectiveness ratio; SD, standard deviation; SE, standard error.

### BCRs and ROIs

We estimate gains in lifetime wages from participation in the program of US$2,729 per child in the group-only arm and US$1,784 in the mixed-delivery arm (Table 5), a difference primarily due to the lower intervention impacts on cognition in the mixed-delivery arm. We estimate future schooling costs of US$35 in the group-only arm and US$23 in the mixed-delivery arm, implying total long-term costs per child of US$176 and US$168, respectively. As a result, Table 5 shows that the benefits of Msingi Bora are estimated to substantially outweigh the associated costs under both delivery models, with a BCR of 15.5 and an ROI of 127% for the group-only delivery model and a corresponding BCR of 10.6 and an ROI of 55% for the mixed-delivery model.

**Table 5:** BCRs and ROI of Msingi Bora.

Delivery model	Group only	Mixed delivery
**Benefits**		
Discounted sum of lifetime earnings per child (US$)	US$13,219	US$13,219
Predicted intervention impact on wages	0.206	0.135
Gains in lifetime earnings per child	**US$2,729**	**US$1,784**
**Costs**		
Present period total costs per child	US$140	US$145
Future schooling costs	US$35	US$23
Total long-term societal costs	**US$176**	**US$168**
**BCR**	15.5 (0.21)	10.6 (0.15)
**ROI (%)**	127% (3.00)	55% (2.23)

Intervention impact on wages calculated as the product of ITT intervention impact estimates from Table 1 and wage returns of 0.397 as described in Table A in S1 Text. Gains in lifetime earnings per child calculated as discounted sum of lifetime earnings multiplied by intervention impact on wages. All costs originally in Kenyan Shillings converted to USD using exchange rate of 1 USD = KSh 101.8 as of January 2020. Present period total costs per child include provider and societal costs (mother’s opportunity costs and venue costs); total long-term societal costs further include future schooling costs. Discounted sum of lifetime earnings adjusts for expected survival probabilities using age life tables from Kenya. Discount rate for age earning profiles is 5%. SEs in parentheses derived using 1,000 Monte Carlo simulations.

BCR, benefit–cost ratio; ITT, intention to treat; ROI, return on investment; SE, standard error.

### Sensitivity analysis

Table 6 shows the calculated values of the key input parameters needed to reach an estimated BCR less than 1 or an estimated ROI less than 0, holding all other values constant. These “extreme values” identify at which point scaling Msingi Bora would become a poor societal investment. For example, to obtain a BCR less than 1 for the group-only delivery model, we would need a discount rate of 17% (as opposed to 5%), holding all other parameters constant at assumed values as listed in column 1. Similarly, for predicted program costs to outweigh benefits under both the group-only delivery and mixed-delivery models (implying a BCR less than 1), our early intervention impacts on cognition would need to nearly completely fade out, from 0.52 SD to 0.03 SD in the long term. Finally, scaling up the group-only version of Msingi Bora would have a negative ROI for the government if the average tax rate was 6.4%, which is less than half our calculated average tax rate.

**Table 6:** One-way sensitivity analysis by intervention delivery model.

	(1)	Group only		Mixed delivery	
		(2)	(3)	(4)	(5)
Input parameter	Benchmark values	BCR < 1	ROI < 0	BCR < 1	ROI < 0
Discount rate	5%	17.0%	9.0%	15.0%	6.6%
Provider costs per child (US$)	US$140 in group and US$145 in mixed	US$2,800	US$365	US$1,700	US$240
Tax rate	14.6%	-	6.4%	-	9.4%
Cognitive wage return	39.70%	2.6%	17.5%	3.5%	25.0%
Cost per additional year of schooling (as % of annual wage)	15.00%	1,100.0%	110.0%	1,100.0%	76.0%
Minimum necessary SD impact on cognition (fade-out)	0.52	0.03	0.23	0.03	0.22

Column 1 shows the benchmark values used to obtain our primary estimates of BCRs and ROIs as presented in Table 5. In columns 2–5, we modify each parameter (row) one at a time holding all others constant to simulate what values are needed to achieve a BCR < 1 or ROI < 0 under the group-only or mixed-delivery models using 1,000 Monte Carlo simulations. Other parameters such as intervention impacts, venue and mother’s opportunity costs, the cost of an additional year of schooling, and lifetime earnings profiles are fixed across all simulations. Minimum necessary impact on cognition refers to what size impact must remain before BCR < 1 or ROI < 0.

BCR, benefit–cost ratio; ROI, return on investment; SD, standard deviation.

In Table B in S2 Text, we simulate new estimates for ICERs, BCRs, and ROIs under the alternative “best case” cost scenario that assumes a more efficient scaled version of the program’s implementation, as well as that allows for socioemotional skills to further influence lifetime wages beyond the impacts of cognition. In the group-only arm, these adjustments increase our ICER to a 0.55 SD improvement in cognition per US$100 investment, with an associated BCR of 22.4 and an ROI of 227%.

## Discussion

We conducted an economic evaluation of 2 potentially scalable and effective group-based ECD parenting interventions targeted to mothers with young children in rural Kenya. We found that the total costs per child were slightly smaller for the group-only model (US$140) than for the mixed-delivery model (US$145) that substituted periodic home visits for group sessions. Coupled with the fact that effect sizes in child outcomes were consistently higher for the group-only delivery model (although not statistically significantly so at traditional levels), these cost estimates imply that the group-only model was also the most cost-effective, with effect sizes per US$100 additional investment of 0.37 SD in cognition, 0.30 SD in receptive language, and 0.16 SD in socioemotional outcomes.

We also found that the long-term economic returns of scaling Msingi Bora could be extremely high if our predicted long-term program impacts hold. Focusing on cognitive outcomes, we find a BCR of 15.5 for the group-only delivery model and 10.6 for the mixed-delivery model, while the average ROIs for a government scaling the program are 127% and 55%, respectively. We would also require very extreme values of our input parameters to have negative ROIs and BCRs less than 1. For example, program costs would outweigh benefits under the group-only model with a discount rate of 17% or if our early impacts in cognition almost entirely fade out in the longer term from 0.52 SD to 0.03 SD.

The small difference in costs across delivery models of US$5 per child is perhaps surprising. However, it is worth remembering that in 12 of 16 sessions (75%), the delivery model was identical across intervention arms, so the only difference in costs comes from the 4 review sessions. In addition, even though CHVs in the mixed-delivery arm spent an average of 27 additional hours traveling to and delivering each review session as home visits, when priced at current local wage rates, the additional hours spent did not translate into large differences in monetary costs.

Our group-only delivery model remains highly cost-effective when we compare our ICERs based on a health provider’s perspective to those we calculate for 2 recent effectiveness studies from Pakistan and India reporting unit costs for similar parenting interventions targeted to children aged 0 to 3 from LMICs and focused on similar outcomes of children’s cognitive and language development [13,14]. We consider only provider costs simply because no earlier studies accounted for societal costs in their analyses, and we adjust all costs to 2020 US$ in purchasing power parity (PPP) terms to facilitate comparisons. Under this criteria, for Msingi Bora, we estimate an ICER of 0.176 SD improvement in cognition per PPP$100 investment. For the Lady Health Worker (LHW) Program from Pakistan, we estimate a lower ICER for cognitive outcomes of 0.148 SD improvement in cognition in similar 2020 US$PPP terms, based on a reported monthly cost per child of US$4.15 (in 2012 US$) in the responsive stimulation arm [13], which was the study arm with the largest impacts on cognition [37], and assuming that each child was treated for 24 months. However, for language outcomes, their program had a relatively larger ICER of 0.17 SD improvement per PPP$100 compared to our study’s estimated ICER of 0.14 SD improvement in language per PPP$100. It is worth noting that their program costs are at least partially lowered by the fact that their interventions were integrated within the existing infrastructure of government health services with already highly trained personnel, costs that were excluded from their analysis under a provider costing perspective. We imagine that our training costs, which are roughly one-third of our total provider costs, may be more realistic in similar settings where existing infrastructure and personnel are not on par with Pakistan’s LHW program [4]. Furthermore, their study does not account for private opportunity costs to participants or delivery agents during their 33-month program as we do.

Another recent effectiveness study conducted in Odisha, India compared group meetings versus individual home visits over 2 years and similarly found group visits to be much more cost-effective for a relatively similar sample to ours [14]. This study also did not account for private opportunity costs of participants or delivery agents. The total provider costs for group meetings were US$76 per child for the 2-year intervention, which translates to US$265 in 2020 PPP terms, implying a cost-effectiveness ratio of 0.106 SD improvement in cognition per PPP$100 investment and 0.114 SD in language. However, these reported costs per child are based on the assumed number of children that a scaled version of this intervention would be able to cover if facilitators worked full time, which is roughly 9 times higher than the actual number of children invited to the intervention. If we similarly assume that each CHV can work full time to conduct 8 group sessions per week, a scaled version of Msingi Bora using only group visits would cost PPP$89 per child using a societal perspective, where we assume a full-time CHV wage again based on local wage rates. At these assumed costs, we would estimate an ICER of 0.58 SD improvement in cognition per PPP$100 investment and 0.47 SD in language, which again makes our program much more cost-effective despite the inclusion of societal costs, which in a scaled version of Msingi Bora we estimate would account for 61% of the total cost per child. Besides having higher impacts on cognitive and language outcomes, other reasons our program is more cost-effective than the one conducted in India include that our group meetings were nearly twice as large as theirs (they invited 7 to 8 children while our median attendance was 13 children at group meetings), and while 23% of their total costs were allocated to purchase toys and learning materials, our program only used play materials freely available in the home.

Msingi Bora’s estimated BCRs are also comparatively high with the very limited number of related interventions reporting similar estimates (Table 7) including 3 home visitation programs in Latin America [32], an intervention delivered through health center meetings and home visits in Jamaica and other Caribbean countries [38], and a combined day care and home visitation program in Nicaragua [39], all of which used a lower discount rate (3%) than we did in our study (5%). Our BCRs are also high when compared to high-profile home visitation programs evaluated in the United States such as the Nurse-Family Partnership Program, for which recent evaluations report BCRs in the range 1.5 to 5 [40,41]. Furthermore, our BCRs and ROIs only consider the increase in discounted lifetime earnings as benefits of program participation and ignore any potential long-term impacts on other health and social outcomes, and, as such, can be interpreted as likely lower bounds on the true potential societal returns (under the assumption that the program impacts do not fade out in the long term).

**Table 7:** Comparison of BCRs of Msingi Bora with similar ECD programs in LMICs.

Study	Interventions and outcomes	BCR
Berlinski and Schady (2015) [32] (Latin America)	Home visits; modeled costs and returns, using 3% discount rate. Outcomes: child cognitive skills; mother’s employment and wages	3.6 (Guatemala), 2.6 (Colombia), and 3.5 (Chile)
Walker and colleagues (2015) [38] (Antigua, Jamaica, and St. Lucia)	Responsive stimulation videos and group discussion at routine health visits. Received small books and puzzles to use at home. Outcome: Griffith Mental Development Scale. Home visits: adaptation of Jamaica Study Benefits: earnings, extrapolated from the Jamaica Study	5.3 (health center intervention) and 3.8 (home visits)
Lopez Boo and colleagues (2014) [39] (Nicaragua)	BCR is for combined effect of micronutrient supplementation (Sprinkles) and ECD, but effect calculated on the basis of anemia	1.5
**Msingi Bora (2019) (Kenya)**	Responsive parenting and nutrition education intervention delivered by a trained CHV in groups	**Group only: 15.5** (discount rate = 5%)

BCR, benefit–cost ratio; ECD, early childhood development; LMIC, low- and middle-income country.

Our study has numerous strengths related to methods and the economic evaluation itself. To the best of our knowledge, our study is the first economic evaluation of an ECD parenting intervention targeted to children under 3 years of age in SSA. Moreover, our study is unique in comparing the cost-effectiveness of different models of delivery contributing to the debate of potentially scalable models for ECD parenting interventions in low-resource settings. Strengths of our study include adoption of a societal perspective for our CEA that accounts for private opportunity costs to participants and delivery agents, the combination of cost-effectiveness and BCAs to assess outcomes, and the inclusion of sensitivity analyses showing the values at which the program generates negative returns. All of these complement each other to provide policymakers a more comprehensive portrait of private and social costs as well as expected economic returns of our program. Limitations of our analysis include that our BCA necessarily relies on different assumptions of how program impacts translate into long-term benefits and costs using external data sources and that we assume that our early impacts can be sustained in the long term. Future work could address these limitations, but obviously necessitate the passage of time. We also do not account for varying levels of schooling quality across public or private schools that may present different economic opportunities for children enrolled in Msingi Bora, and we do not account for patterns of formal and informal employment or internal migration that may influence the profiles of wages by age we used to estimate benefits. We also examine the economic benefits of enhanced human capital only in terms of increased wages, but we do not include other outcomes that could potentially improve such as labor market participation, share of workers in the informal sector, health, life expectancy, or prosocial behaviors. We may similarly be missing some societal costs in addition to those included. As discussed above, we believe the BCR outcomes can be interpreted as the lower bound of the true societal returns as long as our early impacts are sustained, but if there are large costs incurred to society as a result of intervention-related improvements, leaving these out may modify our BCR and ROI estimates.

Our findings highlight the need for careful cost analyses to accompany evaluations of ECD parenting programs in LMICs to help inform governments and policymakers on the potential returns to society and to the government of investing in such programs. The growing recognition worldwide about the importance of a child’s early years and the potential promise of responsive parenting interventions to improve children’s outcomes are not likely to affect policy decision-making without accompanying information about the cost-effectiveness of such investments. We also believe that cost-effectiveness analyses of ECD parenting programs should account for the private opportunity costs to participants and delivery agents for both ethical and practical reasons: Excluding these costs could create perverse incentives for governments to offload program costs onto users and delivery agents, who are often vulnerable and poor citizens whose burden of time should not be assumed to be free. We hope our results can motivate similar analyses from other ECD parenting programs from LMIC settings.

Our results show that an integrated child development intervention featuring exclusively group sessions delivered by paraprofessional community health workers can be very cost-effective. We find that a mixed-delivery model that substitutes periodic home visits for group sessions comes at a slightly higher cost, albeit with a lower benefit, and thus is less cost-effective for our setting. Our results represent a strong case for exploring pathways to scale similar interventions in low-resource rural settings where many children and families could potentially benefit from ECD parenting programming, and investments could pay significant dividends over time.

## Supporting information

S1 Checklist CHEERS statement. CHEERS, Consolidated Health Economic Evaluation Reporting Standards.
(DOCX)

S1 Text Estimation of wage and schooling returns to cognitive abilities. (DOCX)

S2 Text “Best case” scenario cost analysis. (DOCX)

S1 Data Raw cost data for the Msingi Bora CEA and BCA and does some calculations that are later used in the spreadsheet labeled “BCA exercise”. BCA, benefit–cost analysis; CEA, cost-effectiveness analysis.
(ZIP)

## Abbreviations

BCA: benefit–cost analysis
BCR: benefit–cost ratio
CEA: cost-effectiveness analysis
CHEERS: Consolidated Health Economic Evaluation Reporting Standards
CHV: community health volunteer
ECD: early childhood development
ICER: incremental cost-effectiveness ratio
ITT: intention to treat
KIHBS: Kenyan Integrated Household Budget Survey
KLPS: Kenya Life Panel Survey
LHW: Lady Health Worker
LMIC: low- and middle-income country
NGO: nongovernmental organization
PDV: present discounted value
PPP: purchasing power parity
PSDP: Primary School Deworming Project
ROI: return on investment
SD: standard deviation
SE: standard error
SSA: sub-Saharan Africa
SWAP: Safe Water and AIDS Project
ToT: Training of Trainers
UNICEF: United Nations International Children’s Emergency Fund
WHO: World Health Organization
