# Supplementary material for: Cost-effectiveness and economic returns of group-based parenting interventions to promote early childhood development: Results from a randomized controlled trial in rural Kenya
Source: PLoS Med. 2021 Sep 28;18(9):e1003746. doi: 10.1371/journal.pmed.1003746 (PMC8478245; doi:10.1371/journal.pmed.1003746)
Supplement: S1 Text — (DOCX) [file pmed.1003746.s002.docx]

# Supporting Information S2: Estimation of wage and schooling returns to cognitive abilities

The Kenya Life Panel Survey (KLPS) collects longitudinal data for a representative sample of more than 5,000 individuals participating of the Primary School Deworming Project (PSDP) implemented across 75 primary schools from the Busia District in Western Kenya between 1997-2001. We utilize data on cognitive tests such as the Raven Matrices and vocabulary tests collected in rounds 1-3 (2003-2005, 2007-2009, 2011-2014), as well as data on schooling attainment, earnings when individuals are already collected in round 4 (2018-2019). In our regressions we restrict the sample to individuals assigned to the control group of the PSDP to avoid the inclusion of final outcomes affected by the intervention.

Based on a sample of N=904 individuals from the control group with data on annual earnings in KLPS-4, and on cognitive tests in KLPS-1, KLPS-2, or KLPS-3, we estimate regressions of log-annual earnings on an age-standardized cognitive index controlling for covariates and we predict that a one SD increase in cognition is associated with a 39.7% increase in annual earnings (Table A, column 1). The cognitive index is estimated using latent factor models including data from the Raven’s Progressive Matrices and English language scores. Covariates include parental education, survey wave and month of interview, a female indicator variable, and baseline 1998 school grade fixed effects.

Similarly, based on a sample of N=1,130 individuals with available schooling attainment data in KLPS-4 and cognitive measures in previous rounds, we estimate that a one SD increase in cognition is associated with 1.79 additional years of schooling after controlling for the same covariates (Table A, column 2).

**Table A: Wage and Schooling Returns to Cognition**

|  | (1) | (2) |
| --- | --- | --- |
|  | Log Annual Earnings | Years of Schooling |
| Cognition Index | 0.397 | 1.79 |
|  | (0.058)  p<0.001 | (0.12)  p<0.001 |
| Parents' Average Education | 0.54 | 0.19 |
|  | (0.016)  p<0.001 | (0.03)  p<0.001 |
| Outcome Mean | 6.873 | 9.3 |
| Cognition Mean | -0.014 | -0.014 |
| Number of Observations | 904 | 1130 |

Notes: Standard Errors in parentheses with p-values noted below. Sample restricted to the KLPS deworming control group. Log earnings and years of schooling data obtained combining information from KLPS rounds 3 and 4. The Cognition Index combines data from the Raven's Index rounds 1-3 with Swahili (KLPS-1) and English vocab tests (KLPS-1,2). The Raven's Index is demeaned by age, where the age groups used for demeaning are 18-19, 20-21, 22-23, 24-25, 26-27, 28-29, 30-31, and above 32. Parents' Average Education is the average of the highest years of schooling attained by the parents of the KLPS respondent. Covariates include controls for the survey wave and month of interview, a female indicator variable, and baseline 1998 school grade fixed effects. Observations are weighted to be representative of the original Deworming Project Population in Siaya County, Western Kenya.
