# Supplementary material for: Cost-effectiveness and economic returns of group-based parenting interventions to promote early childhood development: Results from a randomized controlled trial in rural Kenya
Source: PLoS Med. 2021 Sep 28;18(9):e1003746. doi: 10.1371/journal.pmed.1003746 (PMC8478245; doi:10.1371/journal.pmed.1003746)
Supplement: S2 Text — (DOCX) [file pmed.1003746.s003.docx]

## Supporting Information S3: “Best case” scenario cost analysis

In the main text, our analysis takes a conservative approach and includes all costs as they occurred in our project, including all implementation delays and inefficiencies, and even includes costs for venue rentals that technically our project did not pay for; it also assumes an opportunity cost for mothers’ time equal to a full wage rate, despite roughly half of mothers not being employed. As such, our main cost-effectiveness, BCR and ROI results arguably represent a conservative costing scenario. In this alternative “best case” cost scenario, we re-estimate similar cost-effectiveness, BCR and ROI figures, but with a number of simplifying assumptions that allow for potential economies of scale and the benefits of hindsight to avoid such inefficiencies in implementation. In particular, differences under this “best case” costing scenario include that we assume that the same level of supervision could have been attained with only three mentor CHVs instead of four, with one per subcounty. Time use surveys and exit interviews with SWAP supervisors confirm this would have been feasible to achieve the same supervision rate, as in practice we often had two supervisors attend the same session. Under this scenario we further assume they are paid a uniform travel reimbursement of US$25 weekly to supervise sessions outside their own villages. Similarly, unexpected implementation delays caused by unplanned and competing health campaigns using CHVs meant that the actual trial duration was 8 months, though the intervention was originally budgeted and planned for 6 months. Under this “best case” cost scenario, we assume a 6-month implementation phase for all fixed costs (e.g., monthly personnel salaries, transport reimbursements, etc.). Finally, we convert the centralized full-board five-day training sessions to take place in the subcounties to save on lodging costs, which mimics the approach we successfully implemented for the monthly subcounty refresher trainings (this includes US$2.50 transport costs per person per day, US$5 for venue costs per day for each site, and US$5 per person per day for meals). With these changes in assumed costs, as well as allowing for an additive effect of socioemotional returns to wages of 6.4% in addition to the effect of cognitive improvements, we find the cost-effectiveness, BCRs, and ROIs listed in Table B. We consider these to be upper-bound estimates of the true numbers in any scaled model of Msingi Bora.

### Table B: Sensitivity Analysis under “Best Case” costing scenario and incorporating socioemotional returns

|  | Group-only | Mixed-delivery |
| --- | --- | --- |
| 1. Socioemotional returns on wages (assuming 0.064 wage return) & “best case” scenario costs |  |  |
| BCR | 22.4 (0.18) | 16.0 (0.17) |
| ROI | 227.2% (2.59) | 134% (2.54) |
| ICER (additive effect of cognitive and socioemotional ITT) | 0.79 (0.276) | 0.56 (0.157) |

Note: BCR stands for benefit cost ratio, ROI for return on investment, ITT for intention to treat, ICER for incremental cost-effectiveness ratio. Benchmark BCR and ROI values against which these should be compared are in Table 4. We assume an additive effect of cognition and socioemotional impacts for both cost-effectiveness ratios and wage returns. For the latter this is calculated as the 1) product of ITT cognitive impact estimates from Table 1 and a wage return of 0.397 as described in Table A in S2 Text, and 2) the product of ITT socioemotional impact estimates from Table 1 and a socioemotional wage return of 0.064. Total costs per child in this “best case” cost scenario are $95 in the group arm and $101 in the mixed-delivery arm. All BCRs and ROIs assume the benchmark input parameters described in the main text for the discount rate, tax rate, cost per additional year of schooling, and exchange rates. Standard Errors in parentheses are estimated based on 1000 Monte Carlo simulations for BCRs and ROIs. Standard errors for incremental cost-effectiveness ratios obtained from standard errors of intervention impacts using a linear transformation of normally distributed random variables.
